# Variable ventilation improves pulmonary function and reduces lung damage without increasing bacterial translocation in a rat model of experimental pneumonia

**DOI:** 10.1186/s12931-016-0476-7

**Published:** 2016-11-25

**Authors:** Raquel F. de Magalhães, Cynthia S. Samary, Raquel S. Santos, Milena V. de Oliveira, Nazareth N. Rocha, Cintia L. Santos, Jamil Kitoko, Carlos A. M. Silva, Caroline L. Hildebrandt, Cassiano F. Goncalves-de-Albuquerque, Adriana R. Silva, Hugo C. Faria-Neto, Vanessa Martins, Vera L. Capelozzi, Robert Huhle, Marcelo M. Morales, Priscilla Olsen, Paolo Pelosi, Marcelo Gama de Abreu, Patricia R. M. Rocco, Pedro L. Silva

**Affiliations:** 1 Laboratory of Pulmonary Investigation, Carlos Chagas Filho Biophysics Institute, Federal University of Rio de Janeiro, Av. Carlos Chagas Filho, s/n, Bloco G-014, Ilha do Fundão, 21941-902 Rio de Janeiro, RJ Brazil; 2 Laboratory of Cellular and Molecular Physiology, Carlos Chagas Filho Biophysics Institute, Carlos Chagas Filho Biophysics Institute, Federal University of Rio de Janeiro, Av. Carlos Chagas Filho, s/n, Bloco G2-048, Ilha do Fundão, 21941-902 Rio de Janeiro, RJ Brazil; 3 Laboratory of Clinical Bacteriology and Immunology, Federal University of Rio de Janeiro, Rio de Janeiro, RJ Brazil; 4 Carlos Chagas Filho Biophysics Institute, Carlos Chagas Filho Biophysics Institute, Federal University of Rio de Janeiro, Av. Carlos Chagas Filho, s/n, Bloco G2-048, Ilha do Fundão, 21941-902 Rio de Janeiro, RJ Brazil; 5 Laboratory of Immunopharmacology, Oswaldo Cruz Institute – Fiocruz, Rio de Janeiro, RJ Brazil; 6 Department of Pathology, University of São Paulo, Av. Doutor Arnaldo, 455, 01246-903 São Paulo, SP Brazil; 7 Pulmonary Engineering Group, Department of Anesthesiology and Intensive Care Therapy, University Hospital Carl Gustav Carus, Dresden University of Technology, Fetschertsrasse 74, 01307 Dresden, Germany; 8 IRCCS AOU San Martino-IST, Department of Surgical Sciences and Integrated Diagnostics, University of Genoa, Largo Rosanna Benzi 8, 16132 Genoa, Italy; 9 Laboratory of Pulmonary Investigation, Centro de Ciências da Saúde, Carlos Chagas Filho Biophysics Institute, Federal University of Rio de Janeiro, Avenida Carlos Chagas Filho, s/n, Bloco G-014, Ilha do Fundão, 21941-902 Rio de Janeiro, RJ Brazil

**Keywords:** Pneumonia, Variable ventilation, Lung mechanics, Lung damage, Inflammation, Molecular biology

## Abstract

**Background:**

Variable ventilation has been shown to improve pulmonary function and reduce lung damage in different models of acute respiratory distress syndrome. Nevertheless, variable ventilation has not been tested during pneumonia. Theoretically, periodic increases in tidal volume (V_T_) and airway pressures might worsen the impairment of alveolar barrier function usually seen in pneumonia and could increase bacterial translocation into the bloodstream. We investigated the impact of variable ventilation on lung function and histologic damage, as well as markers of lung inflammation, epithelial and endothelial cell damage, and alveolar stress, and bacterial translocation in experimental pneumonia.

**Methods:**

Thirty-two Wistar rats were randomly assigned to receive intratracheal of *Pseudomonas aeruginosa* (PA) or saline (SAL) (*n* = 16/group). After 24-h, animals were anesthetized and ventilated for 2 h with either conventional volume-controlled (VCV) or variable volume-controlled ventilation (VV), with mean V_T_ = 6 mL/kg, PEEP = 5cmH_2_O, and FiO_2_ = 0.4. During VV, tidal volume varied randomly with a coefficient of variation of 30% and a Gaussian distribution. Additional animals assigned to receive either PA or SAL (*n* = 8/group) were not ventilated (NV) to serve as controls.

**Results:**

In both SAL and PA, VV improved oxygenation and lung elastance compared to VCV. In SAL, VV decreased interleukin (IL)-6 expression compared to VCV (median [interquartile range]: 1.3 [0.3–2.3] *vs*. 5.3 [3.6–7.0]; *p* = 0.02) and increased surfactant protein-D expression compared to NV (2.5 [1.9–3.5] *vs*. 1.2 [0.8–1.2]; *p* = 0.0005). In PA, compared to VCV, VV reduced perivascular edema (2.5 [2.0–3.75] *vs*. 6.0 [4.5–6.0]; *p* < 0.0001), septum neutrophils (2.0 [1.0–4.0] *vs*. 5.0 [3.3–6.0]; *p* = 0.0008), necrotizing vasculitis (3.0 [2.0–5.5] *vs*. 6.0 [6.0–6.0]; *p* = 0.0003), and ultrastructural lung damage scores (16 [14–17] *vs*. 24 [14–27], *p* < 0.0001). Blood colony-forming-unit (CFU) counts were comparable (7 [0–28] *vs*. 6 [0–26], *p* = 0.77). Compared to NV, VCV, but not VV, increased expression amphiregulin, IL-6, and cytokine-induced neutrophil chemoattractant (CINC)-1 (2.1 [1.6–2.5] *vs*. 0.9 [0.7–1.2], *p* = 0.025; 12.3 [7.9–22.0] *vs*. 0.8 [0.6–1.9], *p* = 0.006; and 4.4 [2.9–5.6] *vs*. 0.9 [0.8–1.4], *p* = 0.003, respectively). Angiopoietin-2 expression was lower in VV compared to NV animals (0.5 [0.3–0.8] *vs*. 1.3 [1.0–1.5], *p* = 0.01).

**Conclusion:**

In this rat model of pneumonia, VV improved pulmonary function and reduced lung damage as compared to VCV, without increasing bacterial translocation.

**Electronic supplementary material:**

The online version of this article (doi:10.1186/s12931-016-0476-7) contains supplementary material, which is available to authorized users.

## Background

Despite advances in medical care, the prevalence and mortality rates of pneumonia remain relatively high [1]. The cornerstone of pneumonia treatment is antibiotic therapy. However, patients may also require mechanical ventilation to maintain adequate gas exchange and reduce the work of breathing. In fact, pneumonia is a major risk factor for the acute respiratory distress syndrome (ARDS) [2], for which protective ventilation with low tidal volumes has been advocated [3]. However, overdistension and cyclic opening and closure of alveolar units, two of the major mechanisms of ventilator-induced lung injury (VILI), may occur even during protective lung ventilation [4–6].

The use of variable tidal volumes, i.e., variable ventilation, has been shown to improve pulmonary function and reduce lung damage in different experimental models of direct [7–9] and indirect [8, 10] ARDS. Nevertheless, variable ventilation has not been tested during pneumonia. Theoretically, periodic increases in tidal volume (V_T_) and airway pressures might worsen the impairment of alveolar barrier function usually seen in pneumonia [11], and could increase bacterial translocation into the bloodstream [12, 13]. On the other hand, since variable ventilation can recruit the lungs [10] and thereby decrease regional stress and strain, a protective effect against lung damage and bacterial translocation might result.

In the present study, we investigated the impact of variable ventilation on respiratory mechanics, gas exchange, and lung histologic damage, as well as markers of lung inflammation, epithelial and endothelial cell damage, and alveolar stress, in a rat model of pneumonia induced by *Pseudomonas aeruginosa*. We hypothesized that variable ventilation would improve pulmonary function and reduce lung damage without increasing bacterial translocation.

## Methods

### Animal preparation and experimental protocol

Thirty-two Wistar rats (weight, 300–410 g) were anesthetized under spontaneous breathing with 2% isoflurane (Isoforine^®^; Cristália, Itapira, SP, Brazil) and randomly assigned to two groups: 1) Pneumonia (PA, *n* = 16), in which *Pseudomonas aeruginosa* 01 (ATCC27853, 5 × 10^7^ CFU diluted in 200 μL saline) was instilled intratracheally (i.t.) (see Additional file 1 for details of the development of the pneumonia model); and 2) Control, in which 200 μL saline was instilled i.t. (SAL, *n* = 16). Eight animals in the SAL and PA groups were not ventilated (NV) and served as controls for computation of lung damage score, ultrastructural damage score, and molecular biology analyses. After 24 h, animals were premedicated intraperitoneally (i.p.) with 10 mg/kg diazepam (Compaz, Cristália, Itapira, SP, Brazil), followed by 100 mg/kg ketamine (Ketamin-S+, Cristália, Itapira, SP, Brazil) and 2 mg/kg midazolam (Dormicum, União Química, São Paulo, SP, Brazil). An intravenous (i.v.) catheter (Jelco 24G, Becton, Dickinson and Company, New Jersey, NJ, USA) was inserted into the tail vein, and anesthesia induced and maintained with midazolam (2 mg/kg/h) and ketamine (50 mg/kg/h). Following local anesthesia with 2% lidocaine (0.4 ml), a midline neck incision and tracheostomy were performed. A second catheter (PE-50, Becton, Dickinson and Company) was then placed in the right internal carotid artery for blood sampling and gas analysis (Radiometer ABL80 FLEX, Copenhagen NV, Denmark), as well as monitoring of mean arterial pressure (MAP) (Networked Multiparameter Veterinary Monitor LifeWindow 6000 V; Digicare Animal Health, Boynton Beach, FL, USA). A 30-cm-long water-filled catheter (PE-205, Becton, Dickinson and Company) with side holes at the tip, connected to a differential pressure transducer (UT-PL-400, SCIREQ, Montreal, QC, Canada), was used to measure the esophageal pressure (Pes). The catheter was passed into the stomach and then slowly returned into the esophagus; its proper positioning was assessed with the “occlusion test” [14]. Animals were then paralyzed with 2 mg/kg pancuronium bromide i.v. (Cristália, Itapira, SP, Brazil), and lungs mechanically ventilated (Inspira, Harvard Apparatus, Holliston, MA, USA) in volume-controlled ventilation (VCV) mode with V_T_ = 6 mL/kg, respiratory rate = 80 breaths/min, FiO_2_ = 0.4, and positive end-expiratory pressure (PEEP) = 5cmH_2_O. Arterial blood gases and lung mechanics were analyzed (Baseline). SAL and PA groups were then randomly assigned to 2 h of conventional VCV or variable VCV (VV). Conventional ventilation settings were similar to those previously applied (V_T_ = 6 mL/kg, respiratory rate = 80 breaths/min, FiO_2_ = 0.4, and PEEP = 5cmH_2_O). At the end of the experiments (End), arterial blood gases were measured and 20 μL of peripheral blood was sampled for bacterial counts. Animals were killed by exsanguination through the arterial line, and their lungs extracted at PEEP = 5cmH_2_O for light microscopy and molecular biology analyses.

### Variable ventilation

**Fig. 1:**
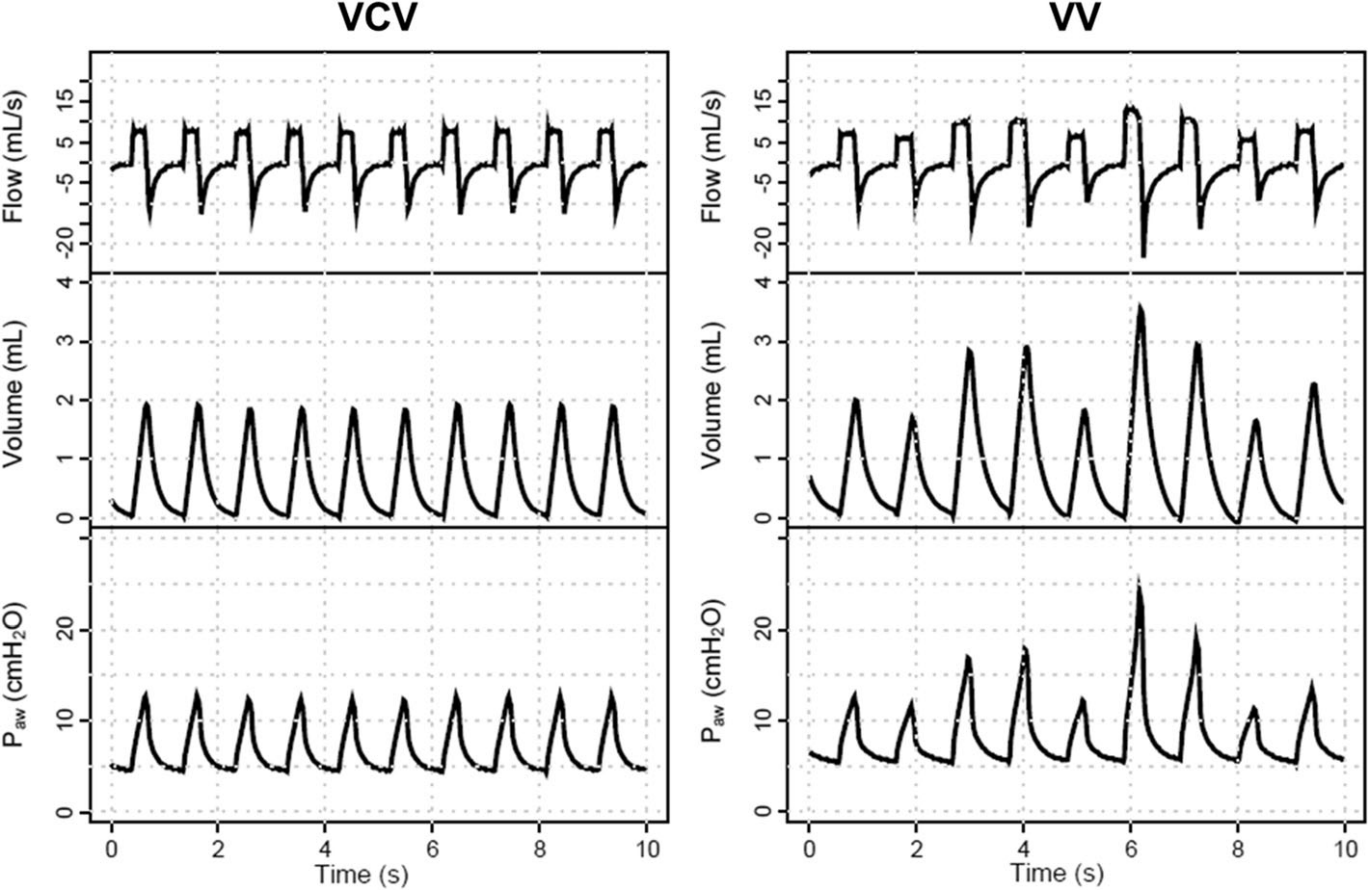
Representative tracings of airway flow, volume, and pressure (P_aw_) during volume controlled ventilation (VCV, *left column*) and variable volume-controlled ventilation (VV, *right column*)

Variable ventilation was applied as described in detail elsewhere [15, 16] Briefly, a sequence of randomly generated V_T_ values (normal distribution, mean = 6 mL/kg, coefficient of variation [CV] = 30%, *n* = 600) was applied in volume-controlled mode using a routine developed by our group (nVentInspira, Dresden, Germany). The sequence continuously looped itself until the end of the experiments. All other mechanical ventilator settings were kept unchanged: mean V_T_ = 6 mL/kg, respiratory rate = 80 breaths/min, FiO_2_ = 0.4, and PEEP = 5cmH_2_O. Figure 1 depicts representative tracings of airflow, volume, and airway pressure (Paw) during conventional ventilation (VCV, left column) and variable ventilation (VV, right column).

### Lung mechanics

Airflow ($\overset{.}{V}$), as well as Paw and esophageal pressure (Pes) were continuously recorded throughout the experiments with a computer running customer-made software written in LabVIEW (National Instruments, Austin, TX) [17]. V_T_ was calculated by digital integration of $\overset{.}{V}$. All signals were amplified in a four-channel signal conditioner (SC-24, SCIREQ, Montreal, QC, Canada). The mechanical properties of the lungs, namely elastance (E_L_) and resistance (R_L_), were calculated by fitting the signals to the equation of motion, according to transpulmonary pressure (P_L_ = Paw−Pes), as shown in Equation 1:

1 $$ {P}_L\;(t)={R}_L \bullet \overset{.}{V}\;(t)+{E}_L \bullet V\;(t)+{P}_{0,L} $$

where P_0_,_L_ is P_L_ at end expiration.

### Lung damage score

The left lung was removed, fixed, and embedded in paraffin. Sections (4 μm thick) were cut and stained with hematoxylin and eosin. A lung damage score based on features commonly seen in pneumonia models was computed [18]. For this purpose, the following histological features were analyzed in the tissue sections: perivascular edema, septal neutrophils, and necrotizing vasculitis. Each feature was scored according to severity, with 0 denoting no effect and 4 denoting maximum severity, and extent, with 0 denoting no appearance and 4 denoting full involvement. The results were calculated as the product of severity and extent of each feature, ranging from 0 to 16, and added to yield the total lung damage score, ranging from 0 to 48.

### Ultrastructural lung damage

To obtain a stratified random sample, three 2 × 2 × 2 mm slices were cut from different segments of the left lung. Ultrathin sections from selected areas were examined and micrographed in a JEOL electron microscope (JSM-6100 F; Tokyo, Japan). In each image (*n* = 15/animal), the following structures were analyzed: 1) type II epithelial cell damage; 2) alveolar-capillary membrane damage; and 3) organelle injury. A procedure similar to that adopted for total lung damage score calculation was used to compute the ultrastructural damage score.

### Blood bacterial counts

Blood samples (20 μL) were seeded in Petri dishes with Tryptic Soy Agar growth medium (Fluka Analytical, St Louis, MO, USA). Manual counts of colony forming units (CFU) were performed after 24 h of incubation at 37 °C.

### Biomarkers of inflammation, alveolar stretch, and cell damage

Quantitative real-time reverse transcription polymerase chain reaction (PCR) was performed to measure biomarkers associated with inflammation (interleukin [IL]-6 and cytokine-induced neutrophil chemoattractant [CINC-1]), type II alveolar cell mechanotransduction (surfactant protein-D [SP-D]), endothelial cell injury (angiopoietin [Ang]-2), and alveolar stretch (amphiregulin). The primers used are described in the online supplement (Additional file 1: Table S1). Central slices of the right lung were cut, collected in cryotubes, flash-frozen by immersion in liquid nitrogen, and stored at − 80 °C. Total RNA was extracted from frozen tissues using the RNeasy Plus Mini Kit (Qiagen, Hilden, Germany), following the manufacturer’s recommendations. RNA concentrations were measured by spectrophotometry in a Nanodrop ND-1000 system (ThermoScientific, Wilmington, DE, USA). First-strand cDNA was synthesized from total RNA using a Quantitec reverse transcription kit (Qiagen, Hilden, Germany). Relative mRNA levels were measured with a SYBR green detection system in an ABI 7500 real-time PCR system (Applied Biosystems, Foster City, California, USA). Samples were run in triplicate. For each sample, the expression of each gene was normalized to the acidic ribosomal phosphoprotein P0 (*36B4*) housekeeping gene [19] and expressed as fold change relative to respective NV animals, using the 2^–∆∆^ Ct method, where ΔCt = Ct_reference gene_ – Ct_target gene_ [20].

### Statistical analysis

Sample size calculation was based on effect estimates obtained from previous studies in rodents using similar ventilator settings [8]. A sample size of eight animals per group would provide the appropriate power (1 − β = 0.8) to identify significant (α = 0.05) differences in respiratory system elastance between VCV and variable ventilation, taking into account an effect size d = 1.38, a two-sided test, and a sample size ratio = 1 (G*Power 3.1.9.2, University of Düsseldorf, Düsseldorf, Germany).

The Kolmogorov-Smirnov test with Lilliefors correction was used to assess the normality of data, whereas the Levene median test was used to evaluate the homogeneity of variances. For the pneumonia model, the Student *t*-test and Mann-Whitney *U* test were used for comparisons of parametric and nonparametric data respectively.

For comparison between conventional and variable ventilations, two-way analysis of variance (ANOVA) followed by Holm-Sidak multiple comparisons was used for analyses of lung mechanics, blood gas exchange, and postmortem parameters (lung damage score, ultrastructural damage score, and blood bacterial counts). Molecular biology analyses were performed using the Kruskal-Wallis test followed by Dunn multiple comparisons within the SAL (NV, VCV, VV) and PA (NV, VCV, VV) groups. Parametric data were expressed as mean ± standard deviation (SD), and nonparametric data, as median (interquartile range). All tests were performed using the GraphPad Prism v6.01 statistical software package (GraphPad Software, La Jolla, California, USA). Significance was established at *p* < 0.05.

## Results

The characterization of the pneumonia model is presented in the online supplement (Additional file 1: Fig. S1, Tables S1, S2, and S3). In PA animals, hemorrhagic areas are present (Additional file 1: Fig. S1), with perivascular edema, neutrophils in alveolar septa, and necrotizing vasculitis (Additional file 1: Table S2). Additionally, an intense inflammatory process characterized by increased cell counts in BALF and blood was observed in PA compared to healthy rats (Additional file 1: Table S3), thus leading to reduced oxygenation (Additional file 1: Table S1). Taken together, those alterations suggest that the pneumonia model was adequate.

MAP was stable throughout the experiment (Table 1). Both in SAL and PA, mean V_T_ was comparable in VV and VCV, whereas CV was higher in VV (Table 1). Compared to VCV, VV reduced E_L_ and increased oxygenation in SAL and PA.

**Table 1:** Respiratory and blood gas-exchange parameters at Baseline and End

		SAL		PA	
Parameter		VCV	VV	VCV	VV
Mean V_T_ (mL/kg)	Baseline	5.8 ± 0.3	6.0 ± 0.1	6.0 ± 0.3	5.9 ± 0.4
	End	5.9 ± 0.6	6.2 ± 0.3	6.0 ± 0.3	6.2 ± 0.4
CV of V_T_ (%)	Baseline	2.5 ± 0.8	2.3 ± 0.4	1.9 ± 0.9	1.9 ± 0.8
	End	1.7 ± 1.0	26.5 ± 1.8****	1.7 ± 0.8	26.6 ± 1.2 ####
E,_L_ (cmH_2_O/mL)	Baseline	3.6 ± 0.5	4.2 ± 0.9	3.9 ± 0.6	4.6 ± 0.7
	End	4.1 ± 0.5	2.5 ± 0.3****	3.8 ± 0.5	2.7 ± 0.2 ##
R,_L_ (cmH_2_O/mL/s)	Baseline	0.19 ± 0.03	0.18 ± 0.03	0.30 ± 0.07**	0.31 ± 0.10
	End	0.19 ± 003	0.16 ± 0.01	0.25 ± 0.07	0.27 ± 0.10
pHa	Baseline	7.4 ± 0.1	7.4 ± 0.0	7.3 ± 0.1	7.3 ± 0.1
	End	7.4 ± 0.1	7.4 ± 0.0	7.4 ± 0.1	7.4 ± 0.0
PaO_2_/FiO_2_	Baseline	372 ± 126	311 ± 83	260 ± 59	285 ± 80
	End	292 ± 78	449 ± 50**	302 ± 117	454 ± 59##
PaCO_2_ (mmHg)	Baseline	40.2 ± 8.0	39.6 ± 6.0	40.2 ± 4.9	42.9 ± 11.1
	End	36.4 ± 10.1	33.9 ± 7.3	37.2 ± 4.8	36.5 ± 8.8
HCO_3_ (mEq/L)	Baseline	23.7 ± 3.1	24.0 ± 2.5	20.8 ± 3.2	20.8 ± 3.2
	End	18.9 ± 4.4	20.0 ± 4.4	21.5 ± 2.6	21.5 ± 2.6
MAP (mmHg)	Baseline	109 ± 24	99 ± 12	96 ± 34	110 ± 27
	End	99 ± 15	110 ± 22	97 ± 28	112 ± 28

Values are mean ± standard deviation (SD) of 8 animals in each group

*Abbreviations*: *SAL-VCV* rats administered intratracheal saline and ventilated with volume-controlled ventilation, *SAL-VV* rats administered intratracheal saline and ventilated with variable ventilation, *PA-VCV* rats administered intratracheal *Pseudomonas aeruginosa* and ventilated with volume-controlled ventilation, *PA-VV* rats administered intratracheal *Pseudomonas aeruginosa* and ventilated with variable ventilation, *V*_*T*_ tidal volume, *CV* coefficient of variation, *E,*_*L*_ dynamic lung elastance, *R,*_*L*_ lung resistance, *pHa* arterial pH, *PaCO*_*2*_ arterial carbon dioxide partial pressure, *PaO*_*2*_ */FiO*_*2*_ arterial oxygen partial pressure divided by fraction of oxygen inspired, *HCO*_*3*_ bicarbonate, *MAP* mean arterial pressure

Comparisons were performed using two-way repeated measures ANOVA followed by the Holm-Šídák post-hoc test (*p* < 0.05). **p < 0.005; ****p < 0.0001 vs SAL-VCV. ##*p* < 0.01; ####*p* < 0.0001 vs PA-VCV

**Fig. 2:**
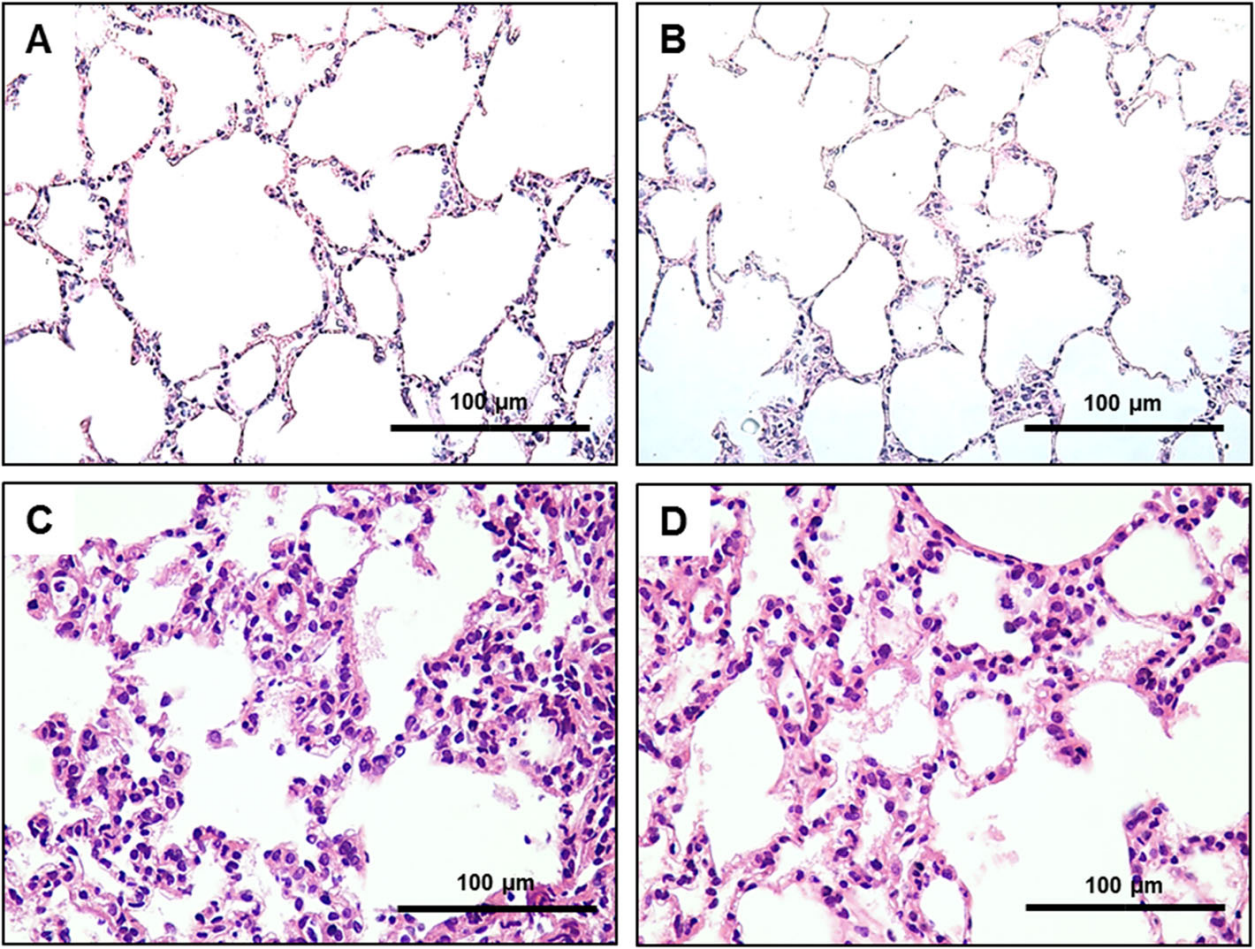
Representative light microscopy images. **a** SAL-VCV: rats administered intratracheal saline and ventilated with volume-controlled ventilation. **b** SAL-VV = rats administered intratracheal saline and ventilated with variable ventilation. **c** PA-VCV = rats administered intratracheal *Pseudomonas aeruginosa* and ventilated with volume-controlled ventilation. **d** PA-VV = rats administered intratracheal *Pseudomonas aeruginosa* and ventilated with variable ventilation. Original magnification: ×400. Scale bar is 100 μm

Light microscopy images of representative animals from each group are shown in Fig. 2. As depicted in Table 2, compared to VCV, VV yielded less perivascular edema, septum neutrophils, and necrotizing vasculitis during PA, but not SAL. Additionally, there was less damage to the lung ultrastructure in VV compared to VCV.

**Table 2:** Lung damage score

	SAL		PA	
Features	VCV	VV	VCV	VV
*Light microscopy*				
Perivascular edema [0–16]	1.5 [1.0–2.0]	1.0 [0.0–2.0]	6.0 [4.5–6.0]*	2.5 [2.0–3.75]#, ‡
Septal neutrophils [0–16]	0.0 [0.0–0.0]	0.0 [0.0–0.0]	5.0 [3.3–6.0]*	2.0 [1.0–4.0]#, ‡
Necrotizing vasculitis [0–16]	1.5 [0.0–2.0]	1.0 [1.0–1.0]	6.0 [6.0–6.0]*	3.0 [2.0–5.5]#, ‡
Total lung damage score [0–48]	2.5 [2.0–3.8]	2.0 [1.0–3.0]	16 [15–18]*	8.0 [5.5–11.3]#, ‡
*Transmission electron microscopy*				
Type 2 epithelial cell damage [0–16]	3 [2–3]	2 [1–2]	6 [4–9]	5 [5–6]
Alveolar capillary membrane damage [0–16]	2 [2–3]	1 [1–2]	9 [4–12]*	5 [5–5]
Organelle injury [0–16]	2 [2–3]	1 [1–2]	6 [6–9]**	6 [4–6]
Total ultrastructural damage score [0–48]	7 [6–9]	5 [3–5]	24 [14–27]***	16 [14–17]##, ‡

Values are median and interquartile range [25–75%] of 8 animals in each group

*Abbreviations*: *SAL-VCV* rats administered intratracheal saline and ventilated with volume-controlled ventilation, *SAL-VV* rats administered intratracheal saline and ventilated with variable ventilation, *PA-VCV* rats administered intratracheal *Pseudomonas aeruginosa* and ventilated with volume-controlled ventilation, *PA-VV* rats administered intratracheal *Pseudomonas aeruginosa* and ventilated with variable ventilation

Comparisons were performed by two-way ANOVA followed by the Holm-Šídák multiple comparison test (*p* < 0.05). *p < 0.05, **p < 0.01, ***p < 0.001 significantly different from SAL-VCV. #*p* < 0.05, ##*p* < 0.01 significantly different from SAL-VV. ‡*p* < 0.05 significantly different from PA-VCV

In SAL, IL-6 expression was lower in VV compared to VCV (Additional file 1: Fig. S2). Moreover, SP-D expression was higher in VV than NV (Additional file 1: Fig. S2).

**Fig. 3:**
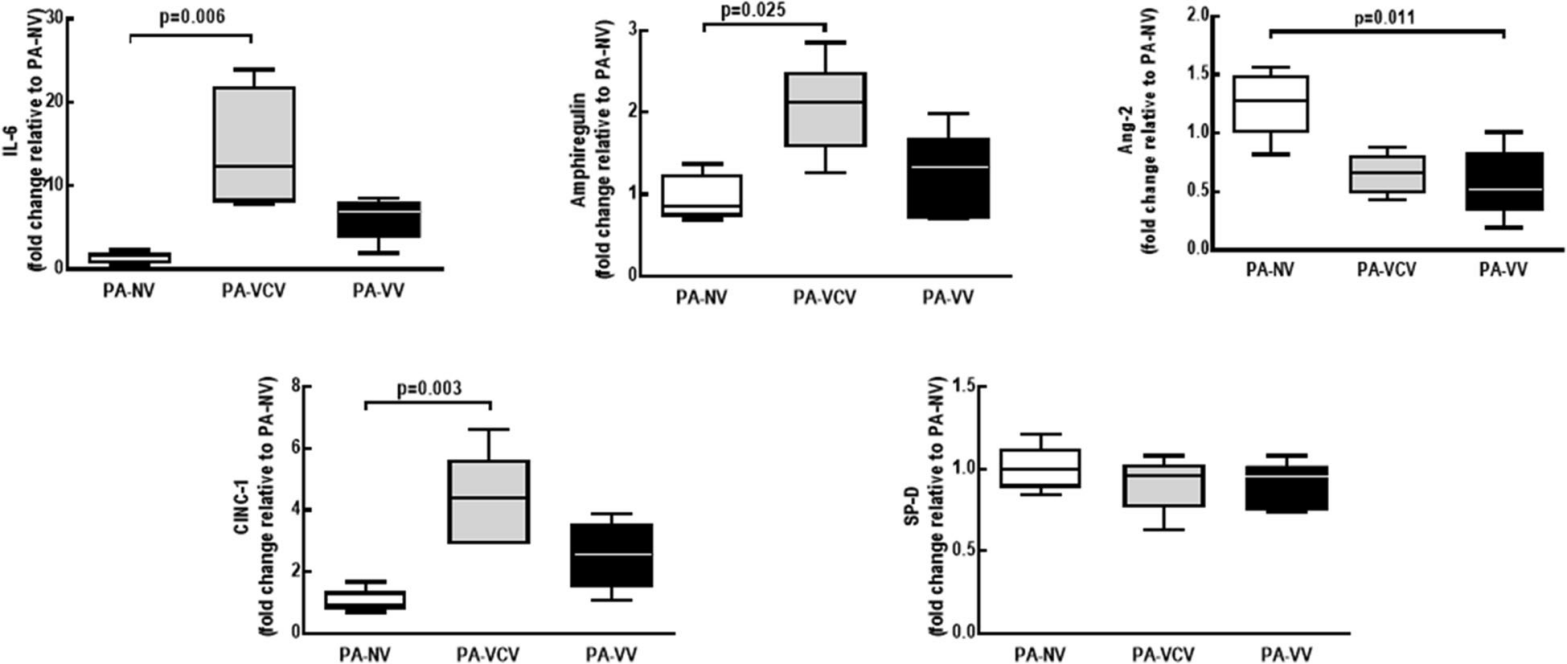
Expression of biological markers. Real-time polymerase chain reaction analysis of biological markers associated with inflammation (IL-6 and CINC-1), alveolar overdistension (amphiregulin), endothelial cell damage (angiopoietin [Ang]-2), and epithelial cell mechanotransduction (surfactant protein [SP]-D). Relative gene expression was calculated as the ratio of average gene expression levels compared with the reference gene (*36B4*) and expressed as fold change relative to non-ventilated (NV) animals with pneumonia (PA). SAL-VCV: rats administered intratracheal saline and ventilated with volume-controlled ventilation; SAL-VV = rats administered intratracheal saline and ventilated with variable ventilation; PA-VCV = rats administered intratracheal *Pseudomonas aeruginosa* and ventilated with volume-controlled ventilation; PA-VV = rats administered intratracheal *Pseudomonas aeruginosa* and ventilated with variable ventilation. Values represent medians and whiskers represent the 10–90 percentile range of 8 animals in each group. Kruskal–Wallis test followed by Dunn’s test for comparisons among groups (*p* < 0.05)

In PA, gene expressions of IL-6, CINC-1, and amphiregulin were higher in VCV, but not in VV, compared to NV. Furthermore, Ang-2 expression was lower after VV compared to NV (Fig. 3).

**Fig. 4:**
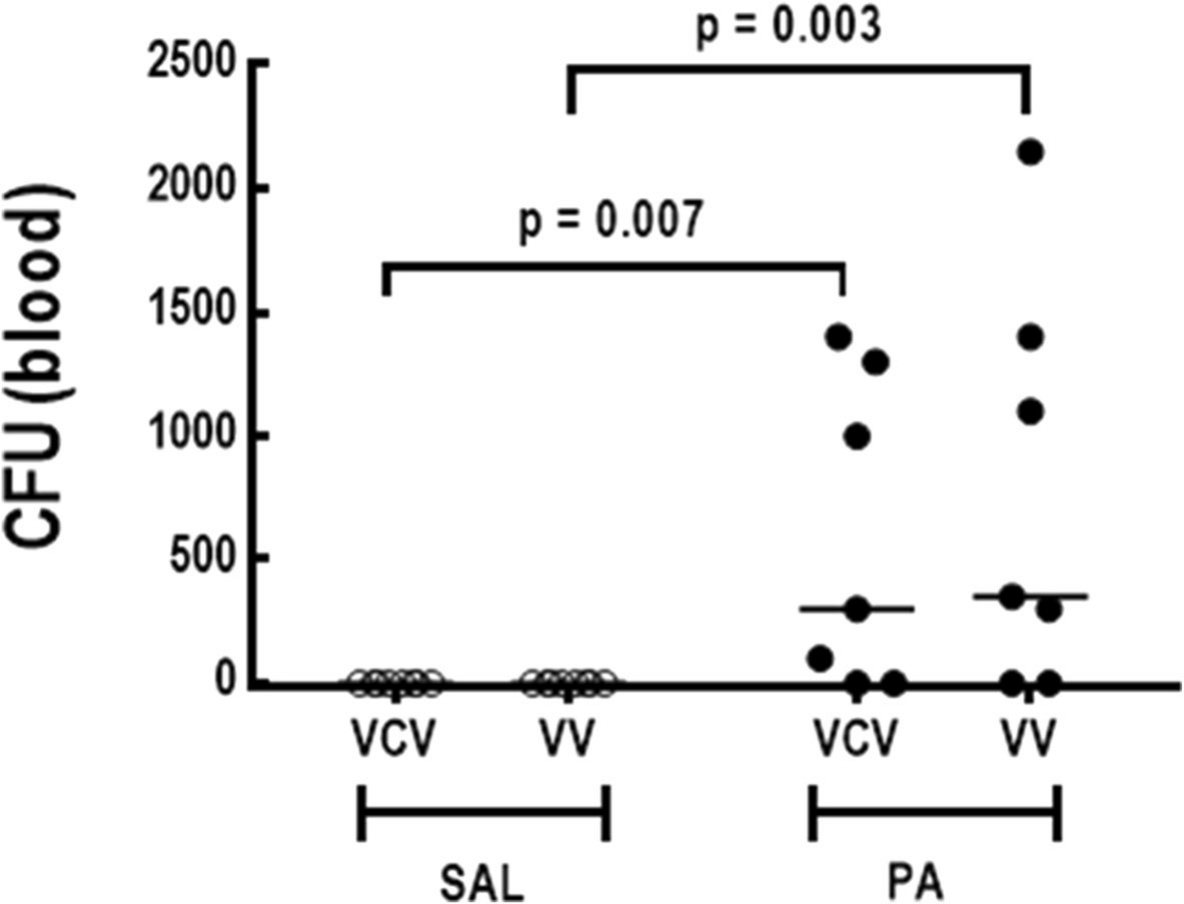
Blood bacterial counts. Each symbol represents individual animals. Black lines are median values of 8 animals in each group. SAL-VCV: rats administered intratracheal saline and ventilated with volume-controlled ventilation; SAL-VV = rats administered intratracheal saline and ventilated with variable ventilation; PA-VCV = rats administered intratracheal *Pseudomonas aeruginosa* and ventilated with volume-controlled ventilation; PA-VV = rats administered intratracheal *Pseudomonas aeruginosa* and ventilated with variable ventilation. Comparisons were performed using two-way ANOVA followed by the Holm–Sidák post-hoc test (*p* < 0.05)

Blood CFU counts were higher in PA than SAL animals (Fig. 4), but values did not differ significantly between VCV and VV, irrespective of group.

## Discussion

The main findings of the present study were: 1) in both SAL and PA, VV improved E_L_ and oxygenation compared to VCV; 2) in SAL, VV was associated with lower IL-6 expression in lung tissue than VCV and increased surfactant protein-D expression compared to NV; 3) in PA, VV reduced perivascular edema, septum neutrophils, necrotizing vasculitis, and ultrastructural lung damage, with no significant difference in blood CFU counts, compared to VCV. Furthermore, mRNA expression of amphiregulin, IL-6 and CINC-1 was higher in VCV, while expression of Ang-2 was lower in VV compared to NV.

A major strength of the present study is that the pathogen chosen, *Pseudomonas aeruginosa*, is a common cause of both community and hospital-acquired pneumonia [21], which is associated with considerable morbidity and mortality [22]. In addition, different aspects of the pneumonia model, including functional, structural, and ultrastructural features, the inflammatory response, and the potential for translocation of bacteria during mechanical ventilation, were characterized in detail (Additional file 1: Figure S1, Tables S1, S2, and S3). We chose a CV of 30% in V_T_ because this level of variability has been shown to improve lung function [8, 23] and reduce lung damage in direct ARDS in rats [8], as well as other species [9, 24–27]. Additionally, controls with i.t. saline instillation were included to allow identification of possible effects that might be specific to the pneumonia model and to exclude the possibility of contamination due to manipulation of the peripheral blood samples. To the best of our knowledge, this was the first study to evaluate variable ventilation in experimental pneumonia.

Our observation that VV improved oxygenation in both PA and SAL, as compared to VCV, can be explained by different factors. First, VV has been shown to promote effective recruitment of atelectatic lungs [10, 28, 29], which seems to be accompanied by redistribution of perfusion to recruited areas [27], improving ventilation/perfusion matching in both SAL and PA. Second, variable V_T_ is able to increase the release of surfactant [30, 31], which could reduce surface tension and further stabilize the lungs, thus improving gas exchange in SAL.

The hypothesis that VV would recruit lungs compared to VCV is supported by the decrease in E_L_. The improvement in E_L_ could partly explain the finding that, in PA, VV led to less perivascular edema, septum neutrophils, and necrotizing vasculitis, which are hallmarks not only of pneumonia [18], but also of VILI [32]. When recruitment occurs, V_T_ is distributed across a larger lung surface area, resulting in decreased regional stress and strain, with less mechanotransduction and biotrauma [33].

This interpretation is supported by the fact that VCV, but not VV, increased the expression of amphiregulin, a marker of pulmonary stretch [34]; IL-6 and CINC-1, which are inflammatory mediators of VILI [35]; and Ang-2, a marker of endothelial integrity [36].

In SAL, VV increased SP-D expression compared to VCV, suggesting that surfactant production was triggered. SP-D plays a central role in pulmonary host defense [37] and migration of peripheral monocyte/macrophages into the lungs [38]. This might explain the reduction in IL-6 with increased SP-D expression in SAL groups (*r* = −0.81, *p* = 0.007). In PA, however, SP-D expression did not differ significantly between VV and VCV. A possible explanation for this difference is that the increased inflammatory response of type 2 epithelial cells due to infection by *Pseudomonas aeruginosa* [39] impaired surfactant production.

We observed that i.t. instillation of *Pseudomonas aeruginosa* increased blood CFU counts in PA compared to SAL. However, among PA animals, CFU blood counts were comparable between VV and VCV. There are different possible explanations for the lack of bacterial translocation during VV in PA. First, the mechanical stress of isolated respiratory cycles may not have exceeded the plasto-elasticity limit of the lung tissue [40], thus preserving the integrity of the alveolar-capillary membrane [41]. Second, lung recruitment likely occurred, reducing volutrauma and atelectrauma, which are intrinsically involved in bacterial translocation during pneumonia [13]. Similar findings have been observed in which PEEP might reduce the risk of ventilation-induced dissemination of bacteria and inflammatory mediators during pneumonia [42, 43].

### Possible clinical implications of study findings

The present study expands the notion that VV is associated with beneficial effects on gas exchange and lung protection in respiratory failure. Since pneumonia is one of the major risk factors for ARDS development [2] and these patients frequently require mechanical ventilation, VV might represent a valuable strategy to improve pulmonary function and reduce lung damage without promoting further injury or bacterial translocation to the blood stream. Furthermore, in patients without lung injury, VV might be useful to prevent deterioration of lung function and increases in inflammatory markers, which could lead to further pulmonary complications. These issues warrant investigation in future experimental and clinical studies.

### Limitations

Some limitations of this study must be noted. First, pneumonia was induced by i.t. instillation of *Pseudomonas aeruginosa*, and our results cannot be extrapolated to other types of pulmonary infection. Nevertheless, in a 10-year retrospective study [21], 45.8% of patients had nosocomial-acquired pneumonia caused by *Pseudomonas aeruginosa*. Furthermore, lungs infected by other pathogens might also benefit from VV-induced responses, e.g., increased production of surfactant. Second, the data presented herein refer to the application of variable ventilation during controlled mechanical ventilation, not assisted ventilation, which might have yielded different results. Third, unlike in clinical settings, PEEP, respiratory rate, and FiO_2_ were kept constant. However, as the main objective was to evaluate VV, confounding factors resulting from changes in ventilator settings were excluded. In this line, the level of PEEP used in the current study, while often used in rats, may not be directly extrapolated to the clinical setting. Nevertheless, it has been estimated that values of PEEP in rats should be multiplied by a factor of 2 to 2.5 [44], when comparing with humans. In our study, this corresponds to 10 to 12.5 cmH_2_O, i.e., a moderate to high PEEP value in humans. Fourth, the observation time was relatively short (2 h of mechanical ventilation), precluding extrapolation of the findings to longer periods of ventilation. Finally, protein levels of biomarkers of VILI were not determined. Instead, we chose to assess expression of biomarker mRNA, because an experimental period of 2 h might not be sufficient to detect differences in protein levels [45–48].

## Conclusions

In the rat model of *Pseudomonas aeruginosa* pneumonia used herein, VV improved pulmonary function and reduced lung damage, without increasing bacterial translocation, compared to VCV.

## Additional file

Additional file 1: Online Supplement. (DOCX 1309 kb)

## Abbreviations

($$ \overset{.}{V} $$): Airflow
36B4: Acidic ribosomal phosphoprotein P0
Ang-2: Angiopoietin
ANOVA: Analysis of variance
ARDS: Acute respiratory distress syndrome
CFU: Blood colony-forming-unit
CINC: Cytokine-induced neutrophil chemoattractant
CV: Coefficient of variation
E_L_: Lung elastance
FiO_2_: Fraction of inspired oxygen
i.v.: Intravenous
IL: Interleukin
MAP: Mean arterial pressure
NV: Non-ventilated
PA: *Pseudomonas aeruginosa*
Paw: Airway pressure
PCR: Polymerase chain reaction
PEEP: Positive end-expiratory pressure
Pes: Esophageal pressure
R_L_: Lung resistance
SAL: Saline
SD: Standard deviation
SP: Surfactant protein
VCV: Volume-controlled ventilation
VILI: Ventilator-induced lung injury
V_T_: Tidal volume
VV: Variable volume-controlled ventilation
