# Supplementary material for: Variable ventilation improves pulmonary function and reduces lung damage without increasing bacterial translocation in a rat model of experimental pneumonia
Source: Respir Res. 2016 Nov 25;17:158. doi: 10.1186/s12931-016-0476-7 (PMC5124241; doi:10.1186/s12931-016-0476-7)
Supplement: Additional file 1: — Online Supplement. (DOCX 1309 kb) [file 12931_2016_476_MOESM1_ESM.docx]

**Online Supplement**

**Variable ventilation improves pulmonary function and reduces lung damage without increasing bacterial translocation in a rat model of experimental pneumonia**

Raquel F de Magalhães^1^ MSc; Cynthia S Samary^1^ PhD; Raquel S Santos^1^ PhD; Milena V de Oliveira^1^ MSc; Nazareth N Rocha^1^ MD, PhD; Cintia L Santos^1^ MD, PhD; Jamil Kitoko^1^ MSc; Carlos A M Silva^4^ PhD; Caroline L Hildebrandt^4^ BS; Cassiano F Goncalves-de-Albuquerque^4^; Adriana Ribeiro Silva^4^ PhD; Hugo C Faria-Neto^4^ MD, PhD; Vanessa Martins da Silva^5^ MSc, PhD; Vera L Capelozzi^5^ MD, PhD; Robert Huhle^6^ MSc; Marcelo M Morales^2^ MD, PhD; Priscilla Olsen^3^ PhD; Paolo Pelosi^7^ MD, FERS; Marcelo Gama de Abreu^6^ MD, PhD; Patricia RM Rocco^1^ MD, PhD; Pedro L Silva^1^ PhD.

^1^Laboratory of Pulmonary Investigation, ^2^Laboratory of Cellular and Molecular Physiology, Carlos Chagas Filho Biophysics Institute; ^3^Laboratory of Clinical Bacteriology and Immunology, Federal University of Rio de Janeiro; ^4^Laboratory of Immunopharmacology, Oswaldo Cruz Institute – Fiocruz; ^5^Department of Pathology, University of Sao Paulo; ^6^Department of Anesthesiology, Dresden University of Technology, Dresden, Germany; ^7^Department of Surgical Sciences and Integrated Diagnostics, University of Genoa, IRCCS AOU San Martino – IST, Genoa, Italy.

.

**MATERIAL AND METHODS**

Pseudomonas aeruginosa *model*

*Pseudomonas aeruginosa* 01 (ATCC27853) obtained from the FIOCRUZ Bacterial Culture Collection were cultured overnight in Luria Broth Base (Invitrogen™ by Life Technologies, Carlsbad, CA, USA) at 37°C to obtain stationary-phase microorganisms. Then, the sample was centrifuged (Eppendorf Centrifuge 5415R, Hamburg, Germany) at 18,188×*g* for 5 minutes and the pellet was washed and resuspended in sterile saline. The sample was analyzed by spectrophotometry and adjusted to the desired dose of 5×10^5^ colony-forming units (CFUs).

Sixteen Wistar rats (weight 300–360 g) were randomly assigned to two groups: 1) Pneumonia (PA), in which *Pseudomonas aeruginosa* (5×10^7^ CFU) was instilled intratracheally; or 2) Control (SAL), in which saline at a comparable volume was administered via the same route. Twenty-four hours after instillation, animals were premedicated with 10 mg/kg diazepam (Compaz, Cristália, Itapira, SP, Brazil), 100 mg/kg ketamine (Ketamin-S+, Cristália), and 2 mg/kg midazolam (Dormicum, União Química, São Paulo, SP, Brazil) by intraperitoneal injection. An intravenous (i.v.) catheter (Jelco 24G, Becton, Dickinson and Company, New Jersey, NJ) was inserted into the tail vein. Following local anesthesia with lidocaine 2% (0.4 mL), a midline neck incision and tracheostomy were made. A polyethylene catheter (PE-50, Becton, Dickinson and Company) was introduced into the right internal carotid artery for blood sampling and gas analysis (Radiometer ABL80 FLEX, Copenhagen NV, Denmark), as well as invasive blood pressure monitoring (Networked Multiparameter Veterinary Monitor LifeWindow 6000 V; Digicare Animal Health, Boynton Beach, FL, USA). A 30-cm-long water-filled catheter (PE-205, Becton, Dickinson and Company) with side holes at the tip and connected to a differential pressure transducer (UT-PL-400, SCIREQ, Montreal, QC, Canada) was used to measure esophageal pressure (Pes). The catheter was passed into the stomach and then slowly returned into the esophagus; its proper positioning was assessed using the “occlusion test” [[1](#_ENREF_1)]. Animals were then paralyzed with 2 mg/kg pancuronium bromide i.v. (Cristália, Itapira, SP, Brazil), and lungs mechanically ventilated (Servo-i, MAQUET, Solna, Sweden) in volume-controlled ventilation (VCV) mode with V_T_ = 6 mL/kg, respiratory rate = 80 breaths/min, FiO_2_ = 0.4, and zero end-expiratory pressure (ZEEP). After stabilization, blood gas exchange and lung mechanics were computed within 3 minutes. Then, 5-µL and 100-mL peripheral blood samples were obtained from the tail vein for bacterial and inflammatory cell counts respectively. Finally, animals were killed by i.v. injection of sodium thiopental 25 mg (Cristália, Itapira, SP, Brazil). The lungs were ligated at the main left bronchus and bronchoalveolar lavage performed in the right lung to obtain bronchoalveolar lavage fluid (BALF) for analyses. The left lung was used for light and ultrastructural microscopy.

*Blood gas exchange*

Arterial blood gas exchange was analyzed in a Radiometer ABL80 FLEX system (Copenhagen NV, Denmark).

*Lung mechanics*

Airflow (­$\dot{V}$), airway pressure (Paw), and esophageal pressure (Pes) were continuously recorded throughout the experiments with a computer running customer-made software written in LabVIEW (National Instruments, Austin, TX) [[2](#_ENREF_2)]. V_T_ was calculated by digital integration of the flow signal. All signals were amplified in a four-channel signal conditioner (SC-24, SCIREQ, Montreal, QC, Canada). The mechanical properties of the lungs, namely elastance (E_L_) and resistance (R_L_), were calculated by fitting the signals to the equation of motion, according to transpulmonary pressure (P_L_ = Paw – Pes), as shown in Equation 1:

 (Eq. 1)

where P_0_,_L_ is P_L_ at end expiration.

*Inflammatory cell profile in blood*

Peripheral blood samples (5 µL) obtained from the tail vein were diluted in 95 µL Türk’s solution for total leukocyte counts in a hemocytometer (Optik Labor, United Kingdom). For differential blood counts, smears were performed with approximately 100 μL of blood sample. The slides were stained with Panótico Rápido LB^®^ (LABORCLIN, Pinhais, PR, Brazil) and differential leukocyte counts performed manually under a light microscope (Olympus BX51, Olympus Latin America, São Paulo, Brazil), determined as percentages of the total leukocyte count obtained in each animal and converted to absolute numbers taking into account the sample dilution.

*Bronchoalveolar lavage fluid*

For BALF, 3 mL of sterile PBS at 37°C was instilled through a polyethylene tracheal tube. This procedure was repeated three times. The recovered content (~70%) was used for analysis. BALF samples were diluted in Türk’s solution for total cell counts in a hemocytometer. Slides obtained by cytospin were stained with Panótico Rápido LB^®^ (LABORCLIN, Pinhais, PR, Brazil) for differential analysis, using a procedure adapted from that described above for leukocyte counts in blood. Additionally, total protein content, as a surrogate of alveolar-capillary membrane permeability, was analyzed by Bradford’s technique [[3](#_ENREF_3)].

*Bacterial counts*

The peripheral blood and BALF samples (20 µL of each) were seeded in Petri dishes containing Tryptic Soy Agar growth medium (Fluka Analytical, St Louis, MO, USA). Manual counts of colony forming units (CFUs) were obtained after 24 hours of storage at 37°C.

*Lung damage score*

After functional data acquisition, heparin (1000 IU) was injected i.v. and the animals killed by exsanguination. The lungs were removed, fixed, and embedded in paraffin. Sections (4 μm thick) were cut and stained with hematoxylin and eosin. A lung damage score based on features commonly seen in pneumonia models was computed [[4](#_ENREF_4)]. For this purpose, the following histological features were analyzed in the tissue: perivascular edema, septal neutrophils, and necrotizing vasculitis. Each feature was scored according to severity, with 0 standing for no effect and 4 denoting maximum severity, and extent (0 denoting no visible change and 4 denoting involvement of the entirety of visible tissue). The results were calculated as the product of severity and extent of each feature, ranging from 0 to 16, and added to yield the total lung damage score, ranging from 0 to 48.

*Ultrastructural lung damage*

To obtain a stratified random sample, three 2×2×2 mm slices were cut from different segments of the left lung. Ultrathin sections from selected areas were examined and micrographed in a JEOL electron microscope (JSM-6100F, Tokyo, Japan). In each image (n=15/animal), the following features were analyzed: 1) type II epithelial cell damage, 2) alveolar-capillary membrane damage; and 3) organelle injury. A procedure similar to that adopted for total lung damage score calculation was used to compute the ultrastructural damage score, which also ranged from 0 to 48.

**Table S1**. Forward and reverse oligonucleotide sequences of target gene primers

| **Gene** | **Primer** | **Primer sequences (5′-3′)** |
| --- | --- | --- |
| IL-6 | Forward | CTC CGC AAG AGA CTT CCA G |
|  | Reverse | CTC CTC TCC GGA CTT GTG A |
| CINC-1 | Forward | TGC ACC CAA ACC GAA GTC AT |
|  | Reverse | TTG TCA GAA GCC AGC GTT CAC |
| SP-D | Forward | AAA TCT TCA GGG CGG CAA A |
|  | Reverse | GGC CTG CCT GCA CAT CTC |
| Amphiregulin | Forward | TTT CGC TGG CGC TCT CA |
|  | Reverse | TTC CAA CCC AGC TGC ATA ATG |
| Ang-2 | Forward | CAG CCA ACC AGG TGA TT |
|  | Reverse | AAG TTG GAA GGA CCA CAT GC |
| *36B4* | Forward | AAT CCT GAG CGA TGT GCA G |
|  | Reverse | GCT GCC ATT GTC AAA CAC |

Primers used in experiments. IL-6, interleukin-6; CINC-1, cytokine-induced neutrophil chemoattractant 1; SP-D, surfactant protein D; Ang-2, angiopoietin-2; *36B4*, acidic ribosomal phosphoprotein P0.

**RESULTS**

**Figure S1.** Lung damage in a rat model of *Pseudomonas aeruginosa*-induced pneumonia.


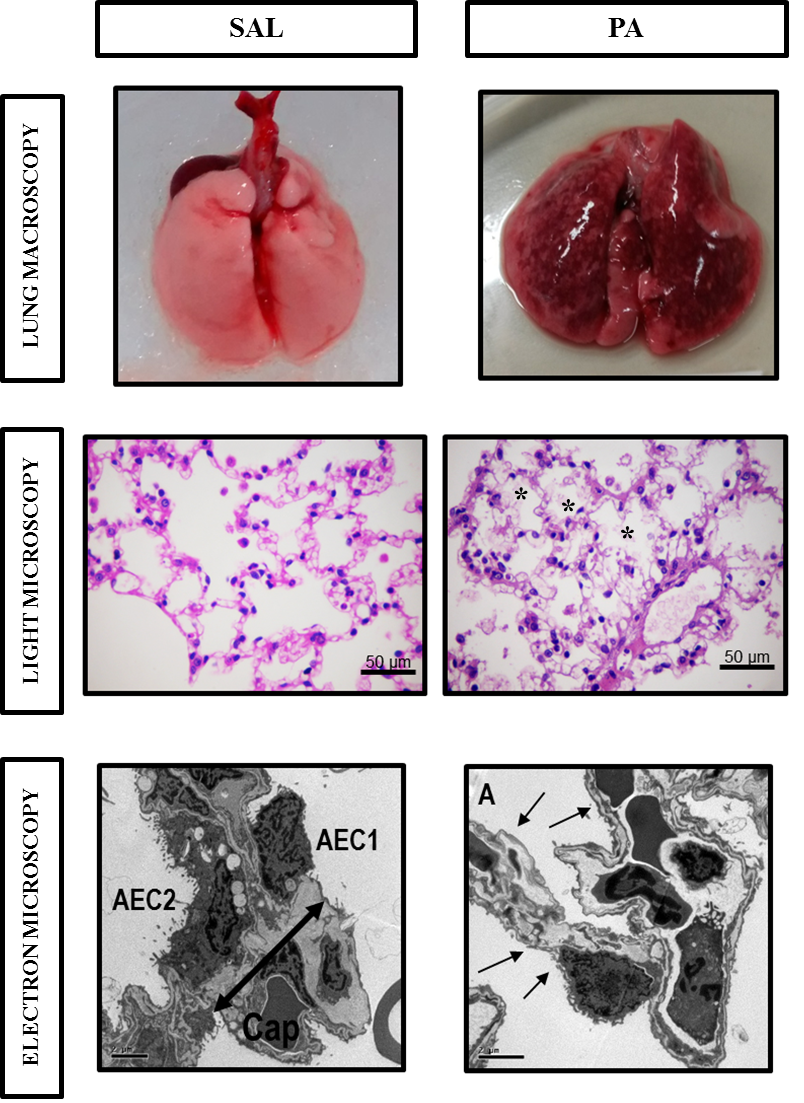


**Figure S1**. AEC1, type 1 epithelial cell; AEC2, type 2 epithelial cell; Cap:, capillary. SAL = rats administered intratracheal saline (Control); PA = rats administered intratracheal *Pseudomonas aeruginosa* (PA). Upper panels: gross appearance showing hemorrhagic areas in PA lungs. Middle panels: photomicrographs of lung parenchyma stained with hematoxylin-eosin (×400). Note increased inflammatory cell infiltration in alveolar septa, alveolar collapse, and edema (asterisk) in PA lungs. Lower panels: photomicrographs of electron microscopy. Double arrowheads: alveolar-capillary membrane. Note the presence of epithelial cell damage (arrows).

**Table S2.** Lung elastance and gas exchange parameters.

|  | E_L_  (cmH_2_O/ml) | pHa | PaO_2_/FiO_2_ | PaCO_2_  (mmHg) | HCO_3_  (mmol/l) |
| --- | --- | --- | --- | --- | --- |
| **SAL** | 4.4 ± 1.3 | 7.4 ± 0.05 | 338 ± 88 | 34.5 ± 2.3 | 22.0 ± 2.4 |
| **PA** | 4.8 ± 1.2 | 7.4 ± 0.04 | 89 ± 22* | 39.6 ± 4.3 | 24.5 ± 2.2 |

Values are mean ± standard deviation of 8 animals in each group at ZEEP. SAL, rats administered intratracheal saline; PA, rats administered intratracheal *Pseudomonas aeruginosa*. E,_L_, dynamic lung elastance; pHa, arterial pH; PaCO_2_, arterial carbon dioxide partial pressure; PaO_2_/FiO_2_, arterial oxygen partial pressure divided by fraction of oxygen inspired; HCO_3_, bicarbonate. Comparisons were performed using the Student *t*-test (p<0.05). *Significantly different from SAL (p<0.05).

**Table S3.** Lung damage score.

|  | **SAL** | **PA** |
| --- | --- | --- |
| *Light microscopy* |  |  |
| Perivascular edema [0-16] | 2 [1.5-2] | 4 [3.5-7]** |
| Septal neutrophils [0-16] | 2 [1.5-5] | 6 [4.5-12]* |
| Necrotizing vasculitis [0-16] | 0 [0-0] | 12 [9-12]*** |
| Total lung damage score [0-48] | 4 [3-7] | 21 [18.5-30]*** |
| *Transmission electron microscopy* |  |  |
| Type 2 epithelial cell damage [0-16] | 0 [0-0] | 12.5 [9-16]*** |
| Alveolar capillary membrane damage [0-16] | 0 [0-0] | 12 [9-12]*** |
| Organelle injury [0-16] | 0 [0-0] | 9 [11-12]*** |
| Total ultrastructural damage score [0-48] | 0 [0-0] | 35 [31-37]*** |

Values are median and interquartile range [25-75%] of 8 animals in each group. SAL, rats administered intratracheal saline; PA, rats administered intratracheal *Pseudomonas aeruginosa*. Comparisons were performed using the Mann-Whitney *U* test. Asterisks denote significant difference from SAL: *p<0.05, **p<0.01, ***p<0.001.

**Table S4.** Inflammatory profile and bacterial count in bronchoalveolar lavage fluid and blood

|  | | | **SAL** | | | **PA** | | |
| --- | --- | --- | --- | --- | --- | --- | --- | --- |
| *Bronchoalveolar lavage fluid* | | |  | | |  | | |
| Total leukocyte count (×10^5^) | | | 3.9 [2.8-4.8] | | | 40.9 [8.5-135.4]*** | | |
| Neutrophils (×10^5^) | | | 0.0 [0.0-0.0] | | | 14.2 [0.4-170.4]*** | | |
| Lymphocytes (×10^5^) | | | 0.1 [0.1-0.2] | | | 0.9 [0.2-6.8]* | | |
| Macrophages (×10^5^) | | | 0.3 [0.2-0.5] | | | 3.2 [2.1-10.2]*** | | |
| Total protein (g/mL) | | | 0.4 [0.4-0.6] | | | 1.2 [0.5-1.4]* | | |
| CFU | | | 0.0 [0.0-0.0] | | | 8,500 [5,150-12,150]* | | |
| *Blood* |  |  | |  |  | |  |  |
| Total leukocyte count (×10^3^/mm^3^) | | | 5.9 [4.4-7.9] | | | 9.3 [5.6-9.9] | | |
| Neutrophils (×10^3^/mm^3^) | | | 0.6 [0.2-2.1] | | | 4.3 [1.9-5.8]** | | |
| Monocytes (×10^3^/mm^3^) | | | 2.9 [1.7-4.0] | | | 3.7 [1.0-4.5] | | |
| CFU | | | 0.0 [0.0-0.0] | | | 575 [175-825]* | | |

Values are median and interquartile range [25-75%] of 8 animals in each group. SAL, rats administered intratracheal saline; PA, rats administered intratracheal *Pseudomonas aeruginosa*. Comparisons were performed using the Mann-Whitney *U* test. Asterisks denote significant difference from SAL: *p<0.05, **p<0.01, ***p<0.001.

**Figure S2.** Molecular biology parameters in SAL animals

**Figure S2**. Expression of biological markers. Real-time polymerase chain reaction analysis of biological markers associated with inflammation (IL-6 and CINC-1), alveolar overdistension (amphiregulin), endothelial cell damage [angiopoietin (Ang)-2], and epithelial cell mechanotransduction [surfactant protein (SP)-D]. Relative gene expression was calculated as a ratio of the average gene expression levels compared with the reference gene (*36β4*) and expressed as fold change relative to non-ventilated (NV) animals administered saline (SAL) intratracheally. VCV, volume-controlled ventilation; VV, variable ventilation. Values represent medians and whiskers represent the 10-90 percentile range of 8 animals in each group. Kruskal–Wallis test followed by Dunn’s test for comparisons among groups (p<0.05).

**References**

1. Baydur A, Behrakis PK, Zin WA, Jaeger M, Milic-Emili J: **A simple method for assessing the validity of the esophageal balloon technique**. *Am Rev Respir Dis* 1982, **126**(5):788-791.

2. Silva PL, Moraes L, Santos RS, Samary C, Ramos MB, Santos CL, Morales MM, Capelozzi VL, Garcia CS, de Abreu MG *et al*: **Recruitment maneuvers modulate epithelial and endothelial cell response according to acute lung injury etiology**. *Crit Care Med* 2013, **41**(10):e256-265.

3. Matute-Bello G, Downey G, Moore BB, Groshong SD, Matthay MA, Slutsky AS, Kuebler WM: **An official American Thoracic Society workshop report: features and measurements of experimental acute lung injury in animals**. *Am J Respir Cell Mol Biol* 2011, **44**(5):725-738.

4. Mizgerd JP, Skerrett SJ: **Animal models of human pneumonia**. *Am J Physiol Lung Cell Mol Physiol* 2008, **294**(3):L387-398.
